# Lifestyle factors, metabolic factors and socioeconomic status for pelvic organ prolapse: a Mendelian randomization study

**DOI:** 10.1186/s40001-023-01148-w

**Published:** 2023-06-07

**Authors:** Hailang Liu, Wei Wu, Wei Xiang, Jingdong Yuan

**Affiliations:** Department of Urology, Wuhan No.1 Hospital, Wuhan Integrated TCM & Western Medicine Hospital, Tongji Medical College, Huazhong University of Science and Technology, Wuhan, China

**Keywords:** Lifestyle factors, Metabolic factors, Socioeconomic status, Mendelian randomization, Pelvic organ prolapse

## Abstract

**Background:**

Previous observational studies have reported that lifestyle factors, metabolic factors and socioeconomic status are associated with the development of female pelvic organ prolapse (POP); however, whether these associations are causal remains unclear. The current study aimed to assess the causal effect of lifestyle factors, metabolic factors and socioeconomic status on POP risk.

**Methods:**

We conducted a two-sample Mendelian randomization (MR) study based on summary-level data from the largest available genome-wide association studies (GWAS) to evaluate whether lifestyle factors, metabolic factors and socioeconomic status are causally related to POP. We used single nucleotide polymorphisms that are strongly associated with exposure at the genome-wide significance level (*P* < 5 × 10^–8^) as instrumental variables from genome-wide association studies. The method of random-effect inverse-variance weighting (IVW) was used as the primary analysis method, supplemented with the weighted median, MR-Egger and the MR pleiotropy residual sum and outlier applied to verify the MR assumptions. Two-step MR was conducted to investigate potential intermediate factors that are on the causal pathway from exposure to POP.

**Results:**

There were associations with POP for genetically predicted waist-to-hip ratio (WHR) (odds ratio (OR) 1.02, 95% confidence interval (CI) 1.01–1.03 per SD-increase, *P* < 0.001), WHR adjusted for body mass index (WHRadjBMI) (OR 1.017, 95% CI 1.01–1.025 per SD-increase, *P* < 0.001) and education attainment (OR 0.986, 95% CI 0.98–0.991 per SD-increase) in the meta-analysis. Additionally, genetically predicted coffee consumption (OR per 50% increase 0.67, 95% CI 0.47–0.96, *P* = 0.03), vigorous physical activity (OR 0.83, 95% CI 0.69–0.98, *P* = 0.043) and high-density lipoprotein cholesterol (HDL-C) (OR 0.91, 95% CI 0.84–0.98 per SD-increase, *P* = 0.049) were inversely associated with POP in the FinnGen Consortium. The mediation analysis showed that the indirect effects of education attainment on POP were partly mediated by WHR and WHRadjBMI, with a mediated proportion of 27% and 13% in the UK Biobank study, respectively.

**Conclusions:**

Our study provides MR evidence of a robust causal association of WHR, WHRadjBMI and education attainment with POP.

**Supplementary Information:**

The online version contains supplementary material available at 10.1186/s40001-023-01148-w.

## Introduction

Pelvic organ prolapse (POP), also called urogenital prolapse, is a disorder that is exclusive to women, especially among those who have given birth and who are postmenopausal. It can affect the anterior vaginal wall, posterior vaginal wall and uterus or apex of the vagina, usually in some combination and thus, it involves the descent of pelvic organs such as the womb (uterus), bladder, bowel and vagina within and outside of the vaginal opening [1]. Loss of vaginal or uterine support in women presenting for routine gynecological care is seen in 43–76% of patients, with 3–6% having descent beyond the hymen [2]. Women in the United States have a 13% lifetime risk of undergoing surgery for POP and it is anticipated that the number of women experiencing POP will increase by approximately 50% by 2050 [3]. In addition, the disorder accounts for 20% of women on the waiting list for major gynecological surgery in the UK [4]. Although POP rarely results in severe morbidity or mortality, it causes vaginal bulge and pressure, voiding dysfunction, defecatory dysfunction and sexual dysfunction, which may adversely affect a woman’s daily activities and quality of life [5]. It has been reported that POP is the leading indication for hysterectomy in postmenopausal women and accounts for 15–18% of procedures in all age-groups [6]. Therefore, identifying the potential causal factors for POP and the direction of their impact could be beneficial for informing prevention strategies.

Epidemiological and observational studies have revealed several possible risk factors for POP, including obesity [7–10], diabetes [9, 10], alcohol consumption [11], coffee consumption [12], smoking [13], physical activity [14], labor [15], education level [13] and hypertension [9, 10]. However, most associations between risk factors and POP are equivocal with inconsistent or contradictory findings across studies [4, 6, 7]. In addition, owing to potential reverse causation and residual confounding issues in observational studies, whether there is an association of the above lifestyle and metabolic factors with POP risk remains undermined. Notwithstanding the perfect performance of randomized controlled trials (RCTs) in causal inference in etiology, it is neither ethical nor feasible to employ RCTs to investigate the influences of lifestyle and metabolic factors on POP. There is a strong demand for alternative methods to infer the potential causality of exposure on outcome.

The Mendelian randomization (MR) design is an emerging genetic method that can strengthen causal inference regarding an exposure–outcome association by leveraging genetic variants as instrumental variables for exposure [16]. The design can minimize residual confounding since genetic variants are randomly allocated during meiosis and therefore not influenced by self-adopted factors or environmental factors that are usually considered as confounders in the association between the exposure and the outcome [17]. Moreover, this method can theoretically diminish reverse causality because the genesis and development of disease cannot modify the germline genotype [17].

Herein, we conducted a two-sample MR investigation to explore the potential causal associations of genetic liability for lifestyle and metabolic factors with POP risk based on the most recent and largest genome-wide association studies (GWAS). Given that education level is strongly correlated with the onset of obesity [18, 19], we performed a two-step MR analysis to investigate the mediating pathway from education attainment to POP via obesity-related phenotypes.

## Methods

### MR design

**Fig. 1:**
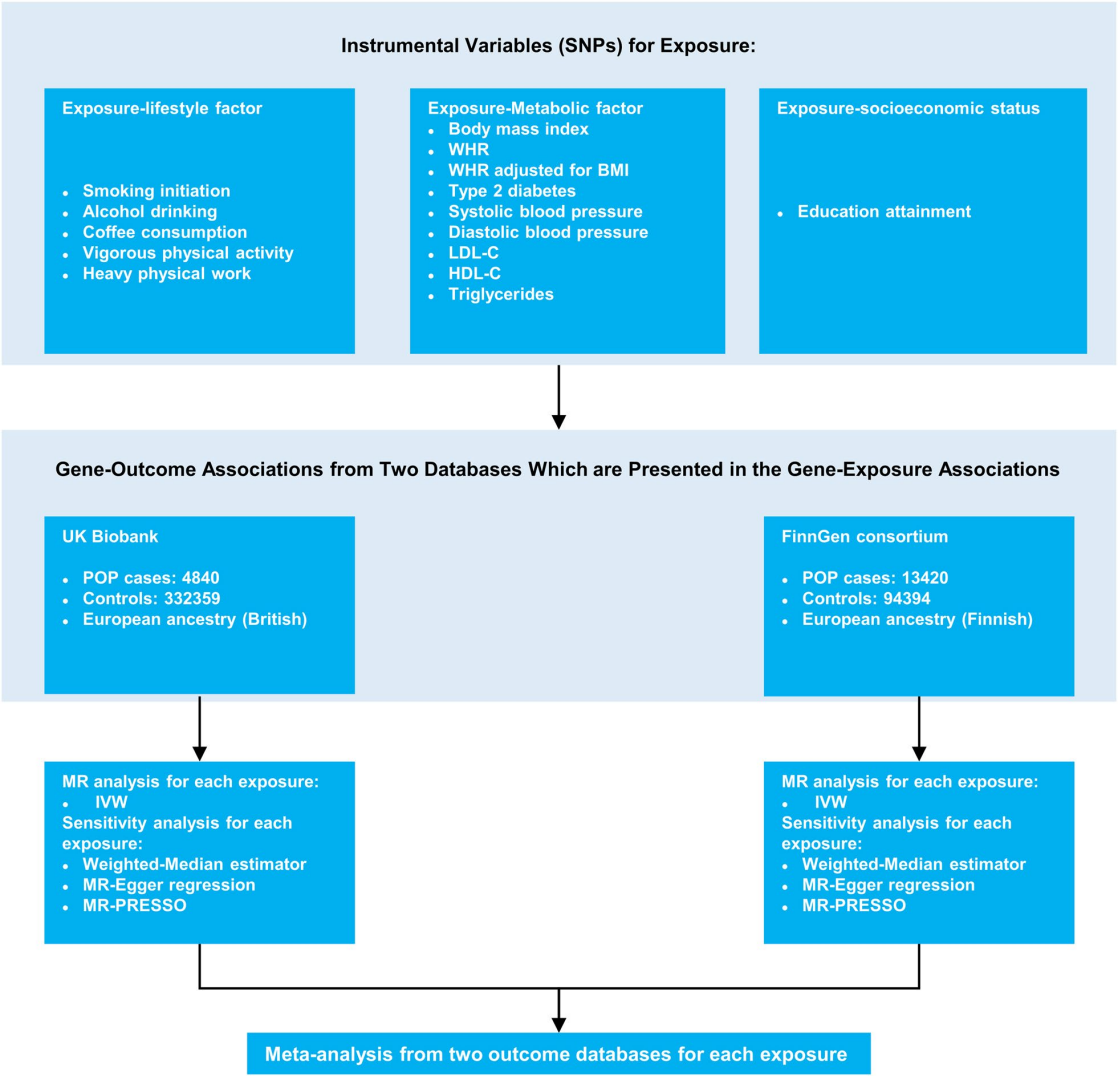
Overview of the Mendelian randomization study design. BMI, body mass index; HDL-C, high-density lipoprotein cholesterol; IVW, inverse-variance weighted; LDL-C, low-density lipoprotein cholesterol; MR, Mendelian randomization; MR-PRESSO, MR-pleiotropy residual sum and outlier; POP, pelvic organ prolapse; SNP, single nucleotide polymorphism; WHR, waist-to-hip ratio

As a genetic variant is usually deemed a proxy for a risk factor in an MR design, the choice of a genetic instrument variable (IV) is particularly important for a successful MR study. MR requires three basic IV assumptions to validate a genetic variant as valid IVs for causal inference: (1) the genetic variant should be robustly associated with the exposure; (2) the genetic variant is not related to potential confounders of the exposure–outcome association; and (3) the genetic variant should have no effect on the outcome other than through the exposure. Additional file 1: Figure S1 shows the three key assumptions of MR analysis. A schematic overview of the present study design is presented in Fig. 1.

### Genetic instrument selection

We mainly selected single-nucleotide polymorphisms (SNPs) associated with metabolic traits and lifestyle factors from the latest and largest sex-specific GWAS studies. SNPs associated with exposures except for vigorous physical activity and heavy physical work (*P* < 5 × 10^–6^) were identified at the genome-wide significance level (*P* < 5 × 10^–8^) and without linkage disequilibrium (*r*^2^ < 0.01 and clumping window > 10,000 kb). Linkage disequilibrium among the SNPs was estimated using the 1000 Genomes European panel as the reference population. We harmonized all variants serving as IVs between the exposure and outcome data by effect allele. Given that a few SNPs were unavailable in the outcome data, we did not find proxies to replace missing single nucleotide polymorphisms. The *F*-statistic was used to assess the strength of IVs. It is a measure of instrument strength that is related to the proportion of variance in the phenotype explained by the genetic variants (*R*^2^), sample size (*N*) and the number of instruments (*k*) by the formula *F* = *R*^2^(*N* − *k* − 1)/*k*(1 − *R*^2^). Generally, an *F-*statistic of > 10 suggests a relatively low risk of bias caused by weak IVs in MR analysis. SNPs associated with body mass index (BMI) [20], waist-to-hip ratio (WHR) [21], waist-to-hip ratio adjusted for BMI (WHRadjBMI) [21], type 2 diabetes [22], smoking initiation [23], alcohol drinking [23], coffee consumption [24], vigorous physical activity [25], heavy physical work, systolic blood pressure (SBP) [26], diastolic blood pressure (DBP) [26], low-density lipoprotein cholesterol (LDL-C) [27], high-density lipoprotein cholesterol (HDL-C) [27] and triglycerides [27] were obtained from corresponding GWAS (Table 1). Smoking initiation was defined as a binary phenotype, which referred to whether an individual had ever smoked cigarettes regularly (current or past smoker) [23]. SNPs associated with WHRadjBMI was used to investigate the BMI-independent effect of WHR [21]. Detailed information about genetic instruments is displayed in Additional file 5: Table S1.

**Table 1:** Detailed information on used studies

Lifestyle factor	Unit	Participants included in analysis	Adjustments	IVs	Sample overlap (%)	PubMed ID
Smoking initiation	SD in prevalence of smoking initiation	1,232,091 European-descent individuals	Age, sex, and the first ten genetic principal components	11	2.5	30643251
Alcohol drinking	SD increase of log-transformed alcoholic drinks/week	941,280 European-descent individuals	Age, sex, and the first ten genetic principal components	6	3.3	30643251
Coffee consumption	50% change	375,833 European-descent individuals	Age, sex, body mass index, total energy, proportion of typical food intake, and 20 genetic principal components	32	6.2	31046077
Vigorous physical activity	≥ 3 versus 0 day/week	98,060 cases and 162,995 controls of European descent	Age, sex, genotyping chip, first ten genomic principal components, and center	15	11.8	29899525
Heavy physical work	Never or rarely; sometimes; usually; always; do not know	288,477 European-descent individuals	–	22	9.6	–
Metabolic factor						
Body mass index	SD	224,459 European-descent individuals	Age, sex, genotyping chip	42	2.9	25673412
WHR	SD	224,459 European-descent individuals	Age, sex, genotyping chip	25	3.1	25673412
WHR adjusted for BMI	SD	224,459 European-descent individuals	Age, sex, genotyping chip	34	3.3	25673412
Type 2 diabetes	One-unit in log-transformed odds	898,130 European-descent individuals	Age, sex, and the first ten genetic principal components	231	0.0	30297969
Systolic blood pressure	10 mm Hg	Up to 1,006,863 European-descent individuals	Age, sex, BMI, genotyping chips	444	3.4	30224653
Diastolic blood pressure	10 mm Hg	Up to 1,006,863 European-descent individuals	Age, sex, BMI, genotyping chips	448	3.6	30224653
LDL-C	SD	188,577 European-ancestry individuals	Age, sex, BMI, genotyping chips	96	7.8	24097068
HDL-C	SD	188,577 European-ancestry individuals	Age, sex, BMI, genotyping chips	122	7.8	24097068
Triglycerides	SD	188,577 European-ancestry individuals	Age, sex, BMI, genotyping chips	71	6.9	24097068
Socioeconomic status						
Education attainment	SD change of education years	765,283 European-ancestry individuals	Age, sex, genotyping chip	404	1.3	35361970

WHR: waist–to-hip ratio; BMI: body mass index; LDL-C: low-density lipoprotein cholesterol; HDL-C: high-density lipoprotein cholesterol; SD: standard deviation; IVs: instrumental variables

### Outcome data sources

We obtained summary-level data for genetic association with POP from the UK Biobank consortium and the FinnGen consortium [DATA FREEZE 7 released on June 1, 2022]. The UK Biobank consortium is an ongoing cohort containing over 500,000 adults at the baseline of recruitment between 2006 and 2010. The study included up to 337,199 individuals (4840 cases and 332,359 controls), after excluding those with sex mismatch, non-Caucasian ethnicity, excess heterozygosity, a low genotype call rate and high relatedness. The seventh release of FinnGen consortium data contains integrated genetic data and disease trajectories from up to 309,154 Finnish biobank participants, 16,962,023 variants and 3095 disease endpoints [28]. For the FinnGen study, we collected data from the R7 release that includes 107,814 Finnish individuals (13,420 cases and 94,394 controls) after removal of those with non-Finnish ancestry, ambiguous gender, excess heterozygosity (± 4 standard deviations) and high genotype missingness (> 5%) [28]. The correlation test was adjusted for sex, age and 10 genetic principal components in both data sources.

### Potential mediators

We included GWAS data sources of potential mediators (obesity-related phenotypes) to investigate potential intermediate factors that are on the causal pathway from exposure to POP. The potential mediators included the following information: BMI from the GIANT consortium with 322,154 individuals of European ancestry [20]; WHR and WHRadjBMI from the GIANT consortium with 210,088 individuals of European ancestry [21]; type 2 diabetes from a meta-analysis of GWAS with 1,339,889 (180,834 cases and 1,159,055 controls) individuals of multi-ancestries (~ 51.1% of European individuals) [22]. Two-step MR was used to investigate the direct and indirect effect of exposure on outcome. First, the effect of exposure on mediator is obtained in a univariable model through regression of the mediator on the exposure. Second, the effect of mediator on outcome is estimated in a univariable model through regression of the outcome on the mediator. Multiplying the two regression estimates from the second stage regression gives the indirect effect of the exposure on the outcome. The mediation proportion can be calculated as the “indirect effect/total effect” using the product of coefficients method [29, 30]. Total effect refers to the causal effect of an exposure on an outcome of interest, including any effect through potential mediators [29, 30].

### Statistical analysis

We used the method of inverse-variance weighting (IVW) with random effects to estimate MR associations between genetic liability to lifestyle factors, metabolic factors and the risk of POP. The IVW method assumes that all SNPs are valid instrumental variables and that the estimates can be interpreted to reflect the total effect of the exposure [16]. It was the primary analysis used to assess causality in this study. Given that the IVW approach only generates an unbiased estimate under the MR assumptions that there is no invalid instrument and horizontal pleiotropy, three sensitivity analysis methods, MR-Egger [31], Mendelian randomization pleiotropy residual sum and outlier (MR-PRESSO) [32] and the weighted median [33], were carried out to examine the robustness of the results and detect horizontal pleiotropy, if any. The heterogeneity of independent SNP effects was assessed by Cochrane *Q* statistics; a *P* value of < 0.05 would be regarded as indicative of significant heterogeneity. The weighted median method specifies that the MR estimates are robust even when up to 50% of the information comes from invalid instrumental variables [33]. MR-Egger regression analysis can detect and correct for directional pleiotropy whereas it compromises power. The *P*-value of the MR-Egger intercept was used to examine the existence of directional pleiotropy. We performed MR-PRESSO analysis to identify possible outliers and generate estimates corrected for outliers [32]. Distortion test results can determine the differences between estimates before and after the removal of outliers. In addition, we used the leave-one-out method to determine which IVs had a significant impact on the estimates. Odds ratios (ORs) and corresponding confidence intervals (CIs) of POP were scaled to a one-standard deviation (SD) increase in prevalence of smoking initiation, a 1-SD increase in WHR, WHRadjBMI and BMI, a 1-unit increase in log OR of type 2 diabetes, a 50% increase in coffee consumption and a 1-SD increase of log-transformed alcoholic drinks/wk. Estimates from the UK Biobank and FinnGen were combined using the fixed-effect meta-analysis method. We selected the *I*^2^ statistic for the assessment of heterogeneity in the meta-analysis. *I*^2^ values of 25%, 50% and 75% were defined as low, medium and high heterogeneity, respectively [34]. All analyses were conducted using R version 4.2.1 and MR analysis was performed using the TwoSampleMR, Mendelian Randomization and MR-PRESSO packages in the R software.

## Results

### MR estimates

Sample overlap was 0–11.8% between the exposures and the outcome data source. Among lifestyle factors, in the primary analyses using IVW, no significant evidence showed that smoking initiation, alcohol drinking and heavy physical work were associated with an increased risk of POP in the UK Biobank study consortium and FinnGen consortium. Univariable MR analysis result from the FinnGen consortium showed a protective causal relationship between coffee consumption and POP. The odds ratio (OR) of POP was 0.67 (95% confidence interval (CI) 0.47, 0.96; *P* = 0.03) for genetically predicted 50% increase in coffee consumption. Nonetheless, this observed association between coffee consumption and POP did not remain in the UK Biobank data and meta-analysis result. Vigorous physical activity was suggestively inversely associated with the risk of POP in the FinnGen consortium. The OR of POP was 0.83 (95% CI 0.69, 0.98; *P* = 0.043) for genetic predisposition to vigorous physical activity. Likewise, we have still not observed the consistent association between vigorous physical activity and POP in the UK Biobank data and meta-analysis result.

**Fig. 2:**
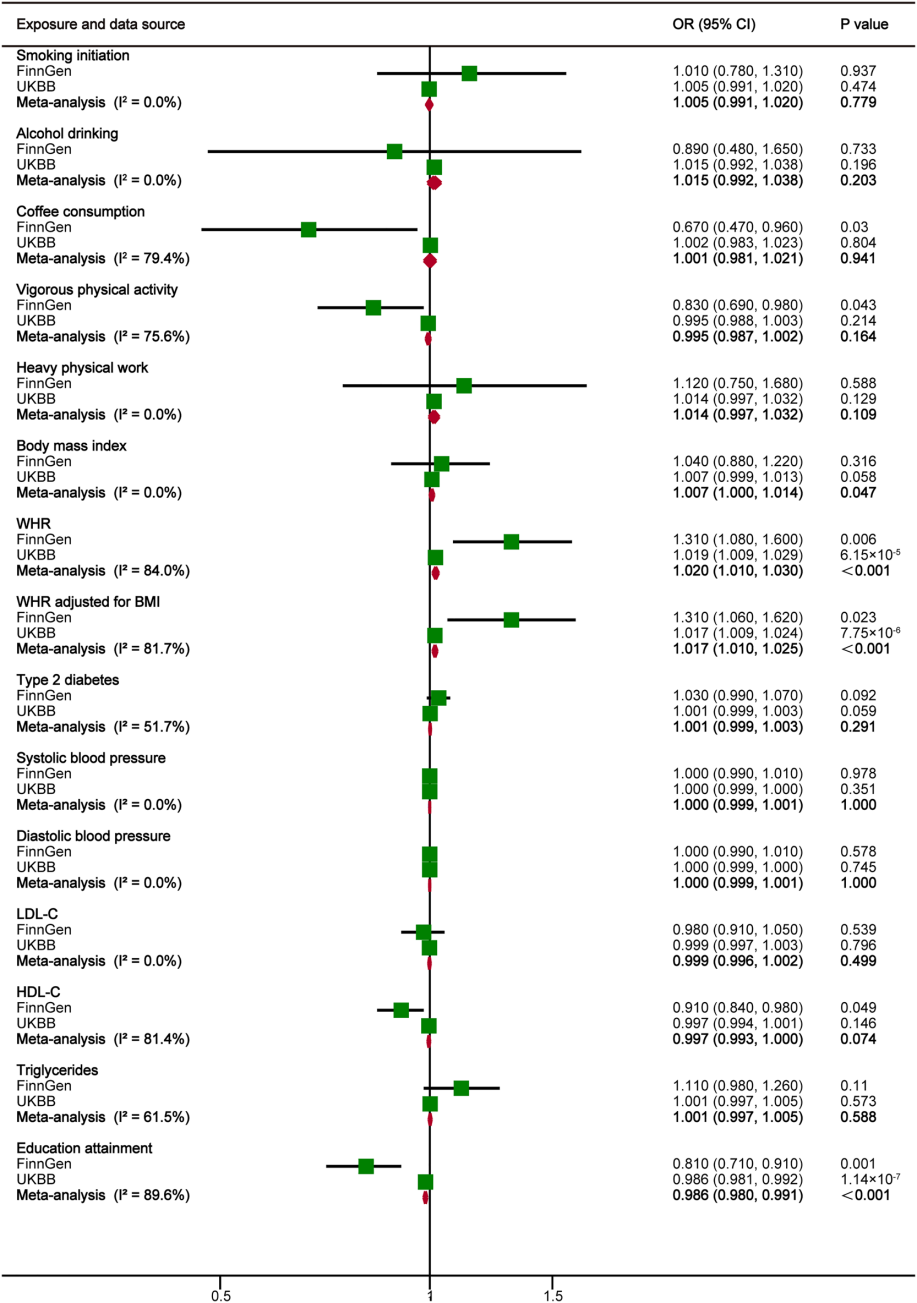
Estimates for the association of genetic liability for lifestyle factors, metabolic factors, and socioeconomic status with risk of pelvic organ prolapse. BMI, body mass index; CI, confidence interval; OR, odds ratio; SNP, single nucleotide polymorphism; HDL-C, high-density lipoprotein cholesterol; LDL-C, low-density lipoprotein cholesterol; WHR, waist-to-hip ratio

Genetically predicted higher WHR and WHRadjBMI was associated with an increased risk of POP in FinnGen consortium data, UK Biobank data and meta-analysis (*P* < 0.05) (Fig. 2). The combined ORs of POP were 1.02 (95% CI 1.01, 1.03; *P* < 0.001) per 1-SD increase in WHR and 1.017 (95% CI 1.01, 1.025; *P* < 0.001) per 1-SD increase in WHRadjBMI. Higher genetically predicted BMI seemed not to be associated with POP in the FinnGen consortium data and the UK Biobank data. It is worth noting that although pooled OR of POP was 1.007 (95% CI 1.000, 1.014; *P* = 0.047) for genetically predicted 1-SD increase in BMI, we should be cautious about interpreting this result. In addition, there was suggestive evidence for the potential benefits of genetically higher HDL-C (OR per 1-SD increase, 0.91, 95% CI 0.84, 0.98; *P* = 0.049) on the risk of POP in the FinnGen consortium data, but not in the UK Biobank data and meta-analysis. No significant associations of POP risk were observed for genetically determined type 2 diabetes, SBP, DBP, LDL-C and triglycerides. We observed a strong protective causal relationship between education attainment and POP in FinnGen consortium data, UK Biobank data and meta-analysis (*P* < 0.05). The ORs of POP were 0.81 (95% CI 0.71, 0.91; *P* = 0.001) in the FinnGen consortium, 0.986 (95% CI 0.981, 0.992; *P* = 1.14 × 10^–7^) in the UK Biobank consortium and 0.986 (95% CI 0.98, 0.991; *P* < 0.001) in the meta-analysis for genetically predicted 1-SD change of education attainment.

### Sensitivity analyses

The observed associations were consistent across sensitivity analyses and between FinnGen consortium data and UK Biobank data overall (Table 2). Moderate-to-high heterogeneity was observed in the analyses for WHRadjBMI, SBP, DBP, HDL-C and triglycerides. We only detected directional pleiotropy for the associations of HDL-C in the UK Biobank consortium and WHR in the FinnGen consortium based on the intercept of the MR-Egger regression model. After removing outlier variants in MR-PRESSO analysis, these associations remained consistent. Additionally, according to the results of leave-one-out analyses, no significant SNPs were driving the relationship between WHR, WHRadjBMI and education attainment and POP (Additional file 2: Figure S2, Additional file 3: Figure S3, and Additional file 4: Figure S4).

**Table 2:** Associations of genetically predicted risk factors with pelvic organ prolapse in MR sensitivity analyses

	SNPs	Cochrane’s *Q*	*F*-statistic	Weighted Median		MR-Egger		MR-PRESSO		*P*_pleiotropy_	*P*_distortion test_
				OR (95% CI)	*P*	OR (95% CI)	*P*	OR (95% CI)	*P*		
UK Biobank											
Lifestyle factor											
Smoking initiation	11	178.50	705	1.002 (0.986–1.018)	0.756	1.011 (0.929–1.102)	0.795	1.005 (0.991–1.020)	0.490	0.886	NA
Alcohol drinking	6	1.83	429	1.020 (0.995–1.046)	0.116	1.024 (0.990–1.059)	0.298	1.015 (0.992–1.038)	0.196	0.552	NA
Coffee consumption	32	53.09	38	1.007 (0.983–1.031)	0.588	0.982 (0.944–1.023)	0.392	1.002 (0.983–1.023)	0.804	0.262	NA
Vigorous physical activity	15	19.77	335	0.994 (0.985–1.003)	0.169	0.982 (0.967–0.997)	0.036	0.995 (0.988–1.003)	0.214	0.074	NA
Heavy physical work	22	26.44	39	1.014 (0.992–1.036)	0.219	1.004 (0.956–1.055)	0.866	1.014 (0.997–1.032)	0.129	0.679	NA
Metabolic factor											
Body mass index	42	51.37	113	1.006 (0.997–1.015)	0.18	1.015 (0.997–1.034)	0.112	1.007 (0.999–1.013)	0.058	0.329	NA
WHR	25	37.71	117	1.018 (1.006–1.029)	0.003	1.05 (1.001–1.102)	0.058	1.019 (1.009–1.029)	6.15 × 10^–5^	0.222	NA
WHR adjusted for BMI	34	48.73	139	1.014 (1.004–1.023)	0.003	1.032 (1.003–1.062)	0.036	1.017 (1.009–1.024)	7.75 × 10^–6^	0.284	0.677
Type 2 diabetes	231	285.31	509	1.001 (0.997–1.003)	0.643	1.00 (0.996–1.004)	0.836	1.001 (0.999–1.003)	0.059	0.499	NA
Systolic blood pressure	444	642.88	186	1.00 (0.999–1.0002)	0.409	0.999 (0.998–1.00)	0.049	1.00 (0.999–1.0001)	0.351	0.094	0.844
Diastolic blood pressure	448	690.40	171	1.00 (0.999–1.0004)	0.635	0.999 (0.998–1.0004)	0.206	1.00 (0.999–1.0005)	0.745	0.159	0.887
LDL-C	96	134.49	249	0.998 (0.995–1.003)	0.611	0.999 (0.994–1.004)	0.711	0.999 (0.997–1.003)	0.796	0.788	0.09
HDL-C	122	166.64	162	0.998 (0.993–1.003)	0.401	1.003 (0.997–1.009)	0.28	0.997 (0.994–1.001)	0.146	0.022	0.828
Triglycerides	71	107.55	195	0.998 (0.993–1.004)	0.539	0.998 (0.991–1.005)	0.565	1.001 (0.997–1.005)	0.573	0.239	0.956
Socioeconomic status											
Education attainment	404	475.25	62	0.985 (0.978–0.992)	5.27 × 10^–5^	0.996 (0.977–1.016)	0.685	0.986 (0.981–0.992)	1.14 × 10^–7^	0.324	0.801
FinnGen consortium											
Lifestyle factor											
Smoking initiation	11	204.75	705	1.04 (0.74–1.45)	0.824	0.79 (0.22–2.85)	0.733	1.01 (0.78–1.31)	0.939	0.715	NA
Alcohol drinking	6	5.08	429	1.09 (0.50–2.41)	0.817	0.29 (0.06–1.35)	0.189	0.89 (0.48–1.65)	0.733	0.193	NA
Coffee consumption	32	29.50	38	0.63 (0.39–1.02)	0.061	0.63 (0.31–1.27)	0.202	0.67 (0.47–0.96)	0.03	0.818	NA
Vigorous physical activity	14	22.94	335	0.89 (0.75–1.07)	0.233	1.02 (0.71–1.47)	0.925	0.83 (0.69–0.98)	0.043	0.229	NA
Heavy physical work	22	28.17	39	0.95 (0.57–1.58)	0.837	1.05 (0.39–2.78)	0.928	1.12 (0.75–1.68)	0.588	0.882	NA
Metabolic factor											
Body mass index	42	57.20	113	1.19 (0.97–1.45)	0.097	1.18 (0.75–1.87)	0.477	1.04 (0.88–1.22)	0.316	0.545	0.697
WHR	25	30.07	117	1.43 (1.11–1.84)	0.006	6.07 (2.50–14.74)	0.001	1.31 (1.08–1.60)	0.006	0.002	NA
WHR adjusted for BMI	34	76.76	139	1.24 (1.01–1.53)	0.039	1.37 (0.60–3.14)	0.463	1.31 (1.06–1.62)	0.023	0.913	0.115
Type 2 diabetes	231	290.77	509	1.01 (0.96–1.07)	0.612	1.06 (0.97–1.15)	0.227	1.03 (0.99–1.07)	0.092	0.595	0.922
Systolic blood pressure	444	807.48	186	1.00 (0.99–1.01)	0.438	0.99 (0.98–1.01)	0.396	1.00 (0.99–1.01)	0.978	0.346	0.019
Diastolic blood pressure	448	764.48	171	0.99 (0.98–1.01)	0.745	1.00 (0.98–1.03)	0.81	1.00 (0.99–1.01)	0.578	0.934	0.903
LDL-C	96	141.92	249	1.03 (0.94–1.13)	0.551	1.01 (0.91–1.12)	0.796	0.98 (0.91–1.05)	0.539	0.373	NA
HDL-C	122	174.50	162	0.85 (0.76–0.95)	0.003	0.85 (0.74–0.98)	0.038	0.91 (0.84–0.98)	0.049	0.333	0.51
Triglycerides	71	162.83	195	1.11 (0.96–1.28)	0.148	1.10 (0.90–1.35)	0.348	1.11 (0.98–1.26)	0.11	0.938	0.763
Socioeconomic status											
Education attainment	404	488.59	62	0.76 (0.64–0.91)	0.002	0.56 (0.37–0.85)	0.007	0.81 (0.71–0.91)	0.001	0.074	NA

BMI, body mass index; CI, confidence interval; OR, odds ratio; SNP, single nucleotide polymorphism; HDL-C, high-density lipoprotein cholesterol; LDL-C, low-density lipoprotein cholesterol; MR, Mendelian randomization; MR-PRESSO, MR-pleiotropy residual sum and outlier; NA, not available. WHR, waist-to-hip ratio

### Mediation analyses

We conducted a two-step MR analysis to examine the mediating pathway from education attainment to POP via four obesity-related phenotypes, including BMI, WHR, WHRadjBMI and type 2 diabetes. In the first step, IVs for education attainment were used to estimate the causal effect of the exposure on the potential mediators. Among the four potential mediators, we found that high education level was associated with decreased WHR (IVW *β* = − 0.196, 95% CI − 0.268 to − 0.125, *P* = 7.43 × 10^–8^) and decreased WHRadjBMI (IVW *β* = − 0.111, 95% CI − 0.175 to − 0.047, *P* = 7.33 × 10^–4^). In the second step, we estimated the causal effect of the mediators on POP risk. We identified causal evidence for effects of WHR (FinnGen: IVW *β* = 0.273, 95% CI 0.077 to 0.469, *P* = 0.006; UK Biobank: IVW *β* = 0.019, 95% CI 0.009 to 0.028, *P* = 6.15 × 10^–5^) and WHRadjBMI (FinnGen: IVW *β* = 0.269, 95% CI 0.054 to 0.485, *P* = 0.023; UK Biobank: IVW *β* = 0.017, 95% CI 0.009 to 0.024, *P* = 7.75 × 10^–6^) on POP in both the FinnGen consortium and UK Biobank study (Table 3). Finally, we investigated the indirect effect of education attainment on POP via WHR and found that the mediation effects of BMI were − 0.054 in the FinnGen consortium and − 0.004 in the UK Biobank study, with a mediated proportion of 25% and 27%, respectively. The indirect effects of education attainment on POP by WHRadjBMI were − 0.03 in the FinnGen consortium and − 0.002 in the UK Biobank study, with a mediated proportion of 14% and 13%, respectively (Table 3).

**Table 3:** The mediation effect of education attainment on POP via WHR and WHR adjusted for BMI

Mediator	Total effect	Direct effect A	Direct effect B	Mediation effect	Mediated proportion (%)
	*β* (95% CI)	*β* (95% CI)	*β* (95% CI)	*β*	
Primary analysis using POP associated SNPs from the UK Biobank					
WHR	− 0.014 (− 0.066, − 0.008)	− 0.196 (− 0.268, − 0.125)	0.019 (0.009, 0.028)	− 0.004	27
WHR adjusted for BMI	− 0.014 (− 0.066, − 0.008)	− 0.111 (− 0.175, − 0.047)	0.017 (0.009, 0.024)	− 0.002	13
Primary analysis using POP associated SNPs from the FinnGen consortium					
WHR	− 0.215 (− 0.338, − 0.091)	− 0.196 (− 0.268, − 0.125)	0.273 (0.077, 0.469)	− 0.054	25
WHR adjusted for BMI	− 0.215 (− 0.338, − 0.091)	− 0.111 (− 0.175, − 0.047)	0.269 (0.054, 0.485)	− 0.03	14

‘Total effect’ indicates the effect of education attainment on POP, ‘direct effect A’ indicates the effect of education attainment on WHR and WHR adjusted for BMI, ‘direct effect B’ indicates the effects of WHR and WHR adjusted for BMI on POP and ‘mediation effect’ indicates the effect of education attainment on POP through WHR and WHR adjusted for BMI. Total effect, direct effect A and direct effect B were derived by IVW; mediation effect was derived by using the product of coefficients method. All statistical tests were two-sided. *P* < 0.05 was considered significant

BMI, body mass index; CI, confidence interval; WHR, waist-to-hip ratio

## Discussion

The present MR study provided genetic evidence of causality between some lifestyle behaviors and metabolic risk factors and POP, showing that WHR and WHRadjBMI in meta-analysis, coffee consumption and vigorous physical activity in the FinnGen consortium, are independently and causally associated with the risk of POP, while education attainment in meta-analysis and HDL-C in the FinnGen consortium are inversely related to the risk of POP. There is no evidence that smoking initiation, alcohol drinking, heavy physical work, type 2 diabetes, SBP, DBP, LDL-C and triglycerides causally associated with POP. Additionally, we also conducted a mediation analysis to estimate potential mediators and showed that the effect of education attainment on POP risk was partially mediated by WHR and WHRadjBMI.

The association between smoking and risk of POP has not been consistent in observational studies. An observational study involving 906 participants showed that smoking was an independent risk factor for POP, while another case–control study including 662 women referred for pelvic floor dysfunction revealed that smoking was not a significant risk factor for POP [35, 36]. A recent meta-analysis including 14 observational studies observed that smoking was found to be a protective factor for POP [37]. Our study found no MR association of smoking with POP risk in two independent datasets. Theoretically, routinely involved in heavy lifting would result in increases in abdominal pressure and may therefore cause progressive pelvic floor damage over time and accelerate the onset of POP [38]. This had been confirmed in an observational study by Gillor et al. [36], which concluded that heavy lifting was significantly associated with POP (OR 1.71; 95% CI 1.2, 2.4; *P* = 0.046). Nevertheless, our findings were not in line with this cohort, suggesting no causal association between heavy physical work and POP in the FinnGen consortium, the UK Biobank study and the meta-analysis. Evidence from epidemiological and observational studies illustrated that most physical activity would not harm the pelvic floor and facilitate the genesis of POP [14, 39, 40]. Similarly, we found no causal effect of genetically predicted vigorous physical activity (≥ 3 days/week) on POP in the present study.

Review articles concerning the association between obesity and POP have revealed consistent evidence that obesity was significantly related to an elevated risk of POP [7–10, 41]. Additionally, we also found that most patients with POP in our medical center were overweight. The most probable pathogenesis theory of POP among overweight and obese women is that the increase in intra-abdominal pressure induced by high weight would harm the pelvic floor fascia and muscles [42]. BMI, representing overall obesity, has demonstrated that a one-unit increase would result in a 3% increase of symptomatic POP (OR 1.03; 95% CI 1.01, 1.05) in the SWEPOP study [43]. With the risk ratio calculated for categories of BMI that conform to the WHO definitions, a recent meta-analysis pooling 22 observational studies investigated the association between degrees of obesity and POP and reported a risk ratio of at least 1.36 (95% CI 1.20, 1.53) in overweight women and at least 1.47 (95% CI 1.35, 1.59) in obese women [44]. Intriguingly, we did not find the causal effect of BMI on POP in the FinnGen consortium and the UK Biobank study, while the meta-analysis result illustrated that pooled OR of POP was 1.007 (95% CI 1.000, 1.014; *P* = 0.047) for a genetically predicted 1-SD increase in BMI in this study. WHR, representing abdominal obesity, can be used to estimate the fat distribution of our body and help indicate a person’s overall health. We also observed suggestive positive associations between genetically determined WHR and the predisposition to POP. The combined OR of POP was 1.02 (95% CI 1.01, 1.03; *P* < 0.001) per 1-SD increase in WHR. WHRadjBMI is a surrogate measure of abdominal adiposity and can also be used to indicate a person’s overall health [45]. In the present study, IVW MR analysis showed that pooled OR of POP was 1.017 (95% CI 1.01, 1.025; *P* < 0.001) per 1-SD increase in WHRadjBMI, indicating that WHRadjBMI was a possible causal risk factor of POP. Other metabolic traits, including type 2 diabetes, SBP, DBP, HDL-C, LDL-C and triglycerides, are usually closely related to overweight and obesity [46]. We also examined whether these metabolic traits’ play a causal role in the genesis and development of POP. It is noteworthy that only genetically predicted higher HDL-C appears to be associated with POP in the FinnGen consortium (OR per 1-SD increase, 0.91, 95% CI 0.84, 0.98; *P* = 0.049).

Epidemiological studies have concluded that obvious regional differences regarding the prevalence of POP and rural areas exist and seem to be correlated with a high prevalence of POP [13, 47, 48]. In addition, we also observed that most POP patients in our center were from deprived regions and had had low education attainments. Education attainment, an important common socioeconomic trait, determines the level of economic development of an area to a large extent. Therefore, we hypothesized that there must be potent causal relationship between education level and POP. Not surprisingly, IVW MR analysis showed evidence indicating a protective causal effect of education attainment on POP risk (OR 0.986, 95% CI 0.98, 0.991; *P* < 0.001). Considering that education level is tightly correlated with obesity [18, 49, 50], we conducted mediation analysis to investigate whether obesity-related phenotypes played a role between education attainment and POP. In the first MR step, univariable MR suggested inverse causal association between genetically determined education level and WHR or WHRadjBMI. A recent study by Böckerman et al. [51] reported an inverse causal effect of education on WHR (IVW *β* = − 0.004, 95% CI − 0.005 to − 0.003), which is aligned with our findings in the first step with regard to direction and magnitude. The second MR step demonstrated that genetically predicted higher WHR and WHRadjBMI were associated with higher odds of POP. In the FinnGen consortium, we found that an estimated 25% and 14% of the total effect was mediated by WHR and WHRadjBMI, respectively. Similarly, in the UK Biobank study, we found that an estimated 27% and 13% of the total effect was mediated by WHR and WHRadjBMI, respectively. These results indicated that the indirect effects of education attainment on POP were partly mediated by WHR and WHRadjBMI.

Our study has several strengths. The major one lies in the use of an MR design based on large-scale GWAS summary datasets to avoid possible bias caused by residual confounders and reverse causality, which are difficult to exclude completely in traditional observational studies. In addition, we combined data from two independent study populations including a huge number of cases with POP, which strengthened the power of the analysis. The current study was confined to subjects of European ancestry, which diminished the population structure bias, so the findings may be specifically generalizable to the European population. Our study has several limitations. First, differences existed regarding the quality control criteria of GWAS for POP between the UK Biobank and FinnGen. We observed high heterogeneity in the combined effects of education attainment, WHR and WHRadjBMI on POP risk; this difference may result in heterogeneity between causal estimates of association. Second, the limitation of our analyses to populations of European ancestry may lead to reduced reliability when extrapolating our findings to individuals of non-European descent. Finally, the major issue for any MR approach is horizontal pleiotropy that means selected genetic instrument variables influence the risk of outcome not via the exposure but confounders. Although we incorporated a range of sensitivity analyses to prevent confounding affecting our conclusions, this possibility cannot be entirely excluded. Lifestyle factors, metabolic factors and socioeconomic status may influence the risk of POP via other pathways. It will be of great significance to identify potential confounders that can detect and predict clinical outcomes among patients with POP.

In summary, our MR study provided robust genetic evidence for the causal role of education attainment, WHR and WHRadjBMI in the risk of POP development. Mediation effects of WHR and WHRadjBMI in the association between education attainment and POP suggest the important role of the management of overweight and obesity in POP prevention. The inverse associations for genetically predicted coffee consumption, vigorous physical activity and HDL-C warrant validation in well-powered studies.

## Supplementary Information

**Additional file 1: Figure S1.** Directed acyclic graph and key assumptions of MR design. For details of assumptions, please refer to the MR design part in the manuscript.

**Additional file 2: Figure S2.** Forest maps of each SNP’s effect for WHR on POP.

**Additional file 3: Figure S3.** Forest maps of each SNP’s effect for WHRadjBMI on POP.

**Additional file 4: Figure S4.** Forest maps of each SNP’s effect for education attainment on POP.

**Additional file 5: Table S1.** Genetic instruments in relation to pelvic organ prolapse.

**Additional file 6: Table S2.** Characteristics of the genetic instrument variables for exposures in both the FinnGen consortium and UK Biobank.

## Data Availability

Summary statistics of GWAS studies were used in this study. The access of data on the pelvic organ prolapse can be obtained by application to the UK Biobank (https://broad-ukb-sumstats-us-east-1.s3.amazonaws.com/round2/additive-tsvs/N81.gwas.imputed_v3.both_sexes.tsv.bgz) and FinnGen consortium (https://storage.googleapis.com/finngen-public-data-r7/summary_stats/finngen_R7_N14_FEMGENPROL.gz). The GWASs for obesity were provided by the GIANT consortium (https://portals.broadinstitute.org/collaboration/giant/index.php/GIANT_consortium_data_files). The GWASs for type 2 diabetes were provided by the DIAGRAM consortium (http://diagram-consortium.org/). The GWASs for lipid profile were provided by the GLGC consortium (http://www.lipidgenetics.org/). Download links for all public datasets are available in Table 1.
