# Supplementary figures and images for: Lifestyle factors, metabolic factors and socioeconomic status for pelvic organ prolapse: a Mendelian randomization study
Source: Eur J Med Res. 2023 Jun 7;28:183. doi: 10.1186/s40001-023-01148-w (PMC10245500; doi:10.1186/s40001-023-01148-w)

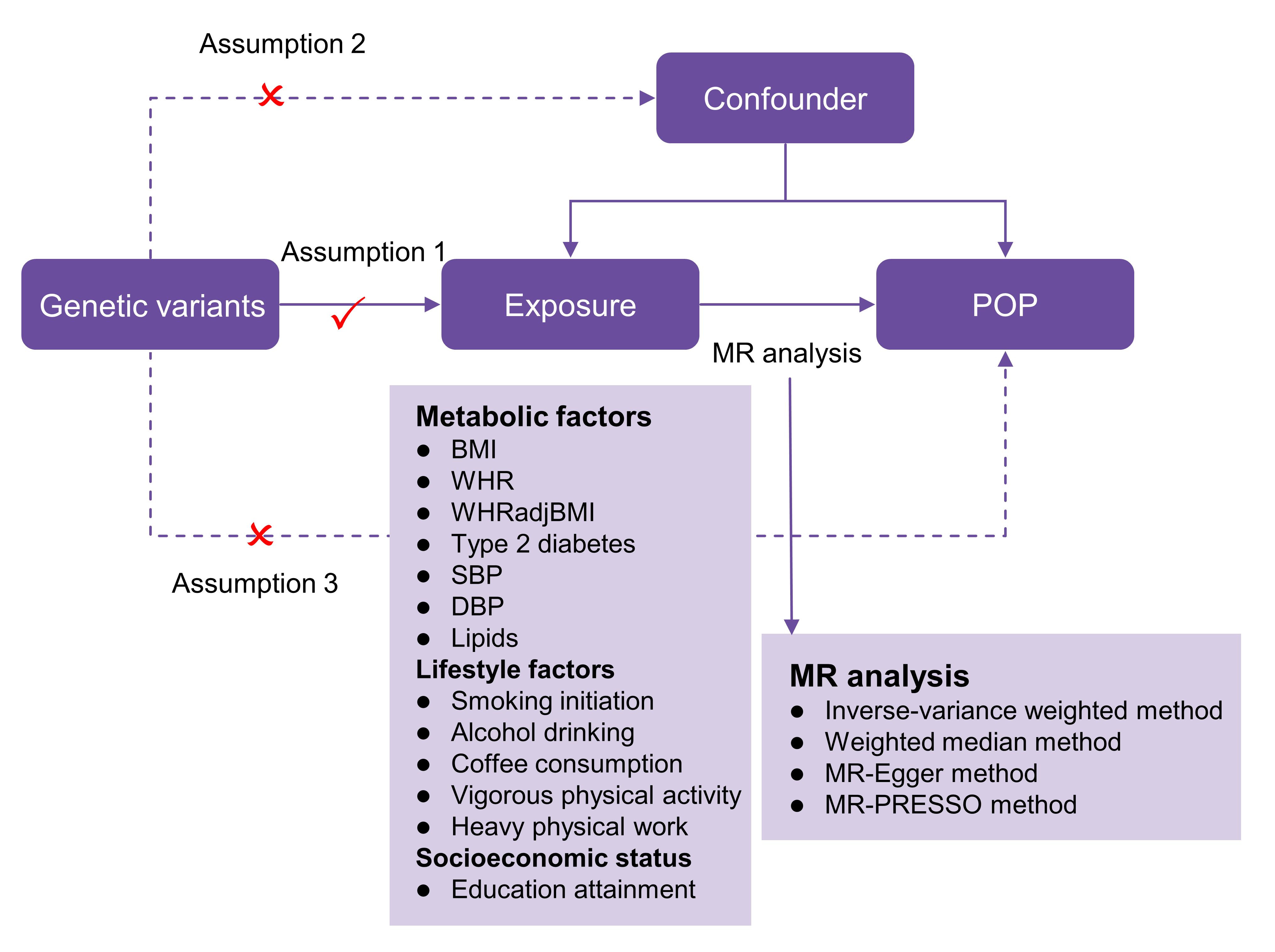

Supplement: Supplementary file 1 — Additional file 1: Figure S1. Directed acyclic graph and key assumptions of MR design. For details of assumptions, please refer to the MR design part in the manuscript. [file 40001_2023_1148_MOESM1_ESM.tif]

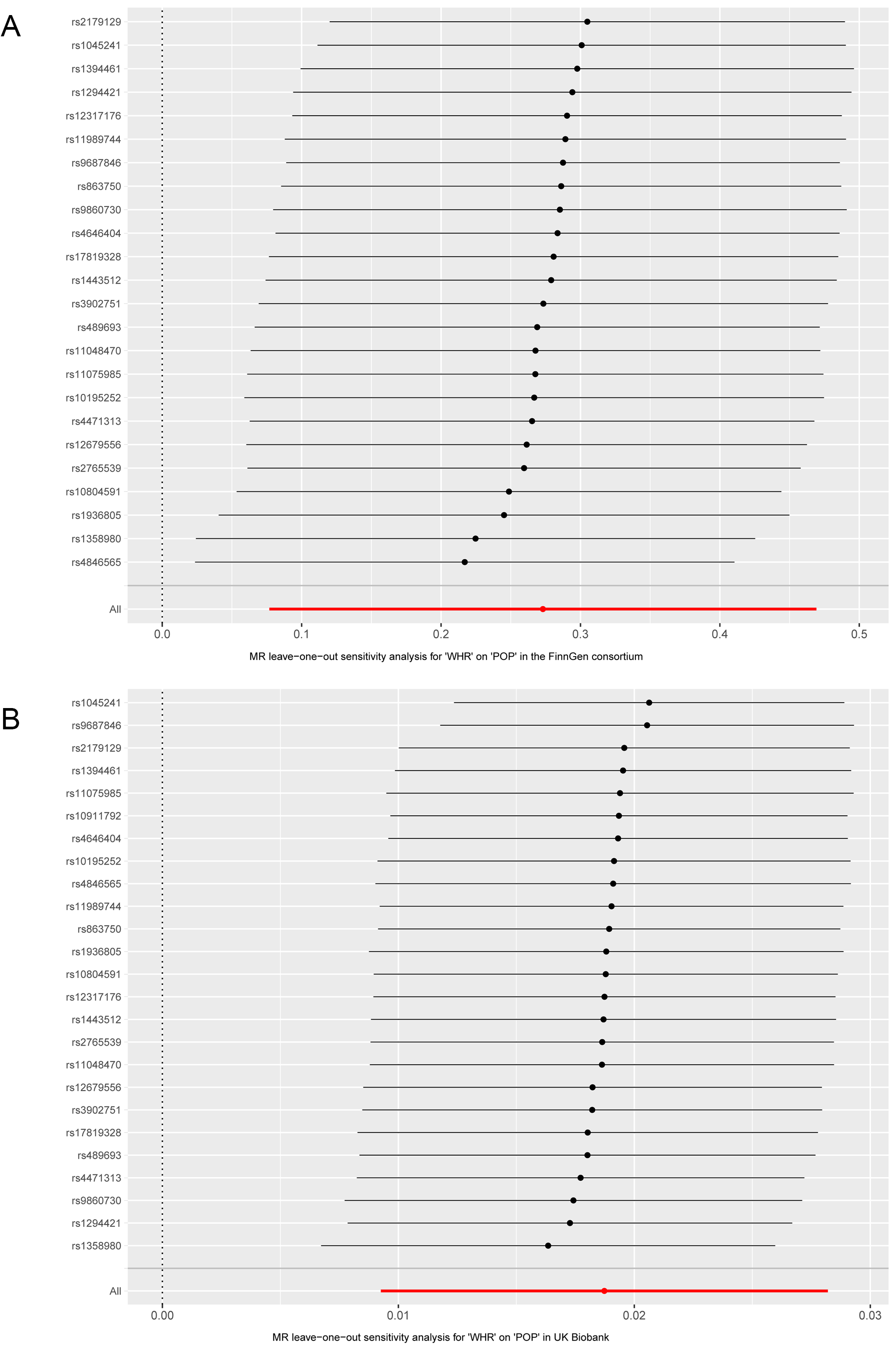

Supplement: Supplementary file 2 — Additional file 2: Figure S2. Forest maps of each SNP’s effect for WHR on POP. [file 40001_2023_1148_MOESM2_ESM.tif]

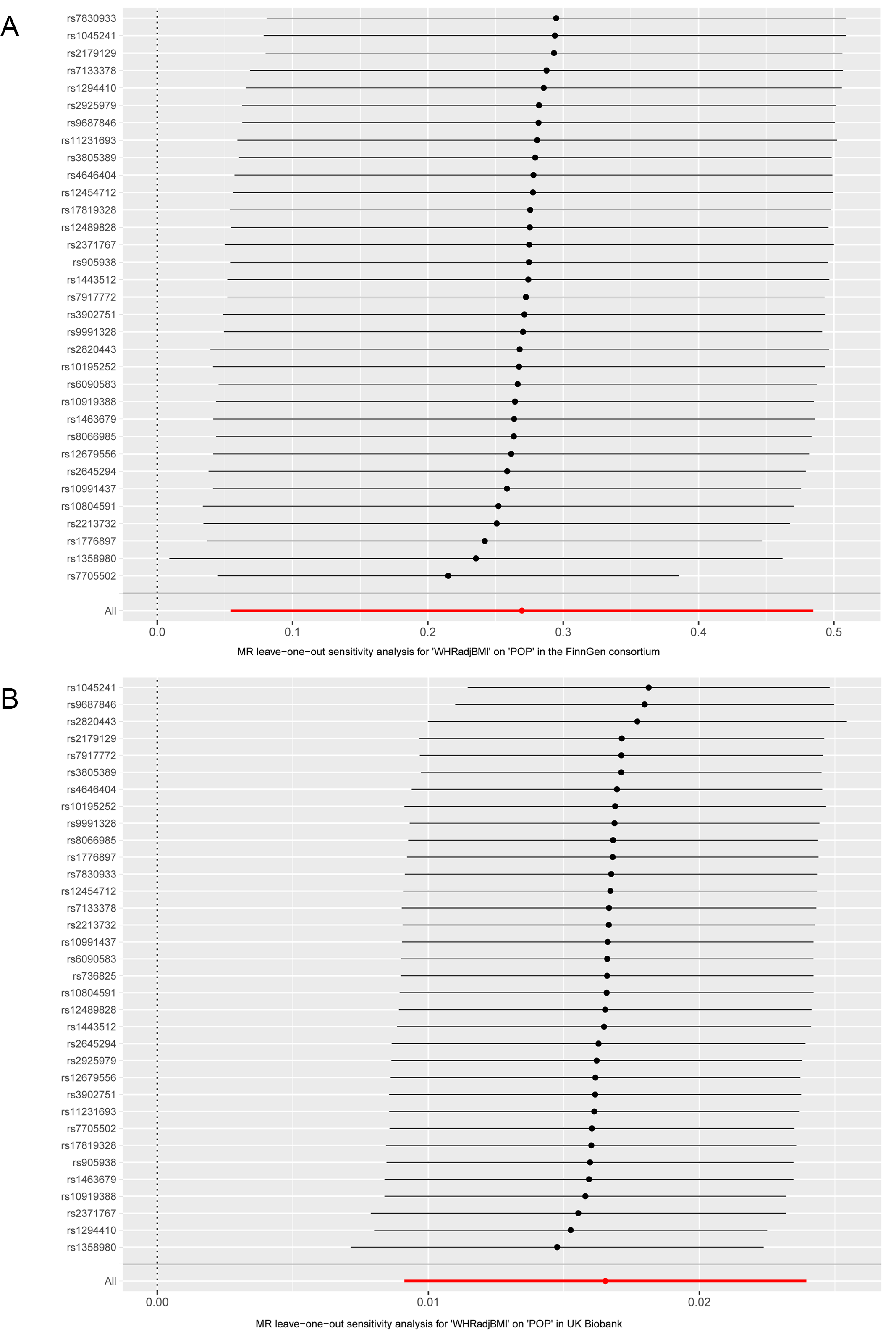

Supplement: Supplementary file 3 — Additional file 3: Figure S3. Forest maps of each SNP’s effect for WHRadjBMI on POP. [file 40001_2023_1148_MOESM3_ESM.tif]

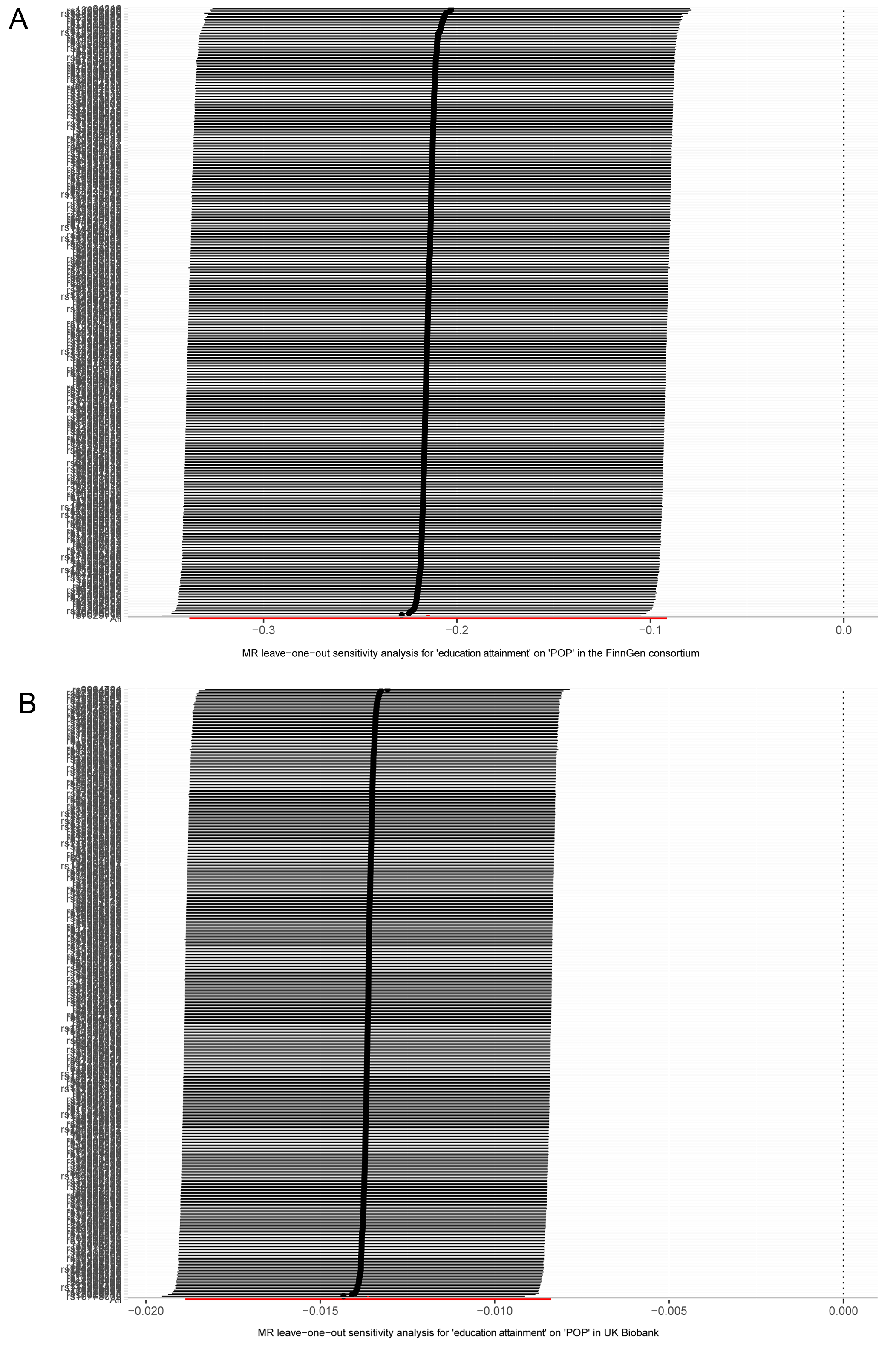

Supplement: Supplementary file 4 — Additional file 4: Figure S4. Forest maps of each SNP’s effect for education attainment on POP. [file 40001_2023_1148_MOESM4_ESM.tif]
